# Supplementary material for: Editors’ perspectives on the peer-review process in biomedical journals: protocol for a qualitative study
Source: BMJ Open. 2018 Oct 18;8(10):e020568. doi: 10.1136/bmjopen-2017-020568 (PMC6196803; doi:10.1136/bmjopen-2017-020568)
Supplement: Supplementary data [file bmjopen-2017-020568supp001.pdf]

Appendix 1. Sample size calculation

```
sampSizeForQual = function(Power, ThemePrevalence, NumInstances) { qnbinom(Power, size =
NumInstances, prob = ThemePrevalence) + NumInstances

}

sampSizeForQual(0.9, 0.1, 2)

[1] 38
```

Code run via R-Fiddle (httm://www.r-fiddle.org)
