# Supplementary material for: Editors’ perspectives on the peer-review process in biomedical journals: protocol for a qualitative study
Source: BMJ Open. 2018 Oct 18;8(10):e020568. doi: 10.1136/bmjopen-2017-020568 (PMC6196803; doi:10.1136/bmjopen-2017-020568)
Supplement: Author's manuscript [file bmjopen-2017-020568.draft_revisions.pdf]

BMJ Open is committed to open peer review. As part of this commitment we make the peer review history of every article we publish publicly available.

When an article is published we post the peer reviewers' comments and the authors' responses online. We also post the versions of the paper that were used during peer review. These are the versions that the peer review comments apply to.

The versions of the paper that follow are the versions that were submitted during the peer review process. They are not the versions of record or the final published versions. They should not be cited or distributed as the published version of this manuscript.

BMJ Open is an open access journal and the full, final, typeset and author-corrected version of record of the manuscript is available on our site with no access controls, subscription charges or pay-per-view fees (http://bmjopen.bmj.com).

If you have any questions on BMJ Open's open peer review process please email info.bmjopen@bmj.com

BMJ Open

The editors’ perspectives on communication practices within the manuscript review process in biomedical journals: Protocol for a qualitative study

Journal:	*BMJ Open*
Manuscript ID	bmjopen-2017-020568
Article Type:	Protocol
Date Submitted by the Author:	09-Nov-2017
Complete List of Authors:	Glonti, Ketevan; Sveuciliste u Splitu, Faculty of Humanities and Social Sciences<br>Hren, Darko; University of Split, School of Humanities and Social Sciences
**Primary Subject Heading**:	Medical publishing and peer review
Secondary Subject Heading:	Qualitative research
Keywords:	peer review, QUALITATIVE RESEARCH, scientific publishing

SCHOLARONE™
Manuscripts

The editors' perspectives on communication practices within the manuscript review process in biomedical journals: Protocol for a qualitative study

Authors: Ketevan Glonti[1, 2, 3], Darko Hren[1]

[1] University of Split, School of Humanities and Social Sciences, Split, Croatia. kglonti@unist.hr, dhren@unist.hr

[2] University Paris Descartes, Paris, France

[3] METHODS Team, Epidemiology and Biostatistics Sorbonne Paris Cité Research Centre, UMR 1153, INSERM.

Corresponding author: Ketevan Glonti, Poljička 35, 21000 Split, Croatia, kglonti@unist.hr

Tel: +385 976324635

Abstract

Introduction: Despite dealing with scientific output and potentially having an impact on the quality of research published, the manuscript peer review process itself has at times been criticised for being 'unscientific'. Research indicates that there are social and subjective dimensions of the peer review process that contribute to this perception, including how key stakeholders - namely authors, editors and peer reviewers - communicate. In particular, it has been suggested that the expected roles and tasks of stakeholders need to be more clearly defined and communicated if the manuscript review process is to be improved. Disentangling current communication practices, and outlining the specific roles and tasks of the main actors, might be a first step towards establishing the design of interventions that counterbalance social influences on the peer review process.

The purpose of this article is to present a methodological design for a qualitative study exploring the communication practices within the manuscript review process of biomedical journals from the journal editors' point of view.

Methods and analysis: Semi-structured interviews will be carried out with editors of biomedical journals between October 2017 and February 2018. A heterogeneous sample of participants representing a wide range of biomedical journals will be sought through purposive maximum variation sampling, drawing from a professional network of contacts, publishers, conference participants, editors associations and snowballing.

Interviews will be thematically analysed following the method outlined by Braun and Clarke. The qualitative data analysis software NVivo v11 will be used to aid data management and analysis.

Ethics and dissemination: This research project was evaluated and approved by the University of Split, Medical School Ethics Committee (2181-198-03-04-17-0029) in May 2017. Findings will be disseminated through a publication in a peer-reviewed journal and presentations during conferences.

For peer review only - http://bmjopen.bmj.com/site/about/guidelines.xhtml

Keywords: Peer review, Biomedical publishing, Scientific journal publishing, Qualitative research

Strengths and limitations of this study

- Qualitative analysis of interview data from a wide range of editors of biomedical journals will allow an in-depth understanding of the communication practices prevailing within biomedical journals.
- Quality assurance will be employed throughout data collection and analysis to ensure traceability and verification.
- Journal editors of a selection of biomedical journals will be interviewed; therefore, research findings cannot directly be extrapolated to all biomedical journals and other scientific fields.

Introduction

Most journals in the biomedical field implement a pre-publication process which primarily involves the interaction of three key stakeholders around an academic research manuscript: journal editors, peer reviewers and authors. This process, typically referred to as 'peer review', is strongly embedded in the field of biomedical publishing and in its broadest sense refers to the evaluation of manuscripts submitted for publication by researchers from the same or related areas of expertise. Thus far, there is no universal definition of 'peer review', and its specific objectives are not clearly defined (1). Concurrently, the roles, tasks and core competencies expected of peer reviewers and editors have not been formally established and both groups operate largely without formal training (2,3).

The peer review process has at times been criticised for being 'unscientific' (4,5). Despite dealing with scientific output that potentially leads to changes in clinical practice, the process itself is not without potential biases - including prestige or association bias, gender bias, confirmation bias, conservatism, bias against interdisciplinary research, publication bias, language bias, and conflict of interest (6,7).

In light of this criticism, there have been several attempts to improve the peer review process and the quality of peer reviewer reports in the biomedical field (8). A recent systematic review evaluating the impact of interventions to improve the quality of peer review for biomedical publications identified 25 strategies that have been implemented, including training interventions; use of checklists (such as Consort (9)); addition of specific experts (i.e. statistical peer reviewers); the introduction of open peer review (i.e., peer reviewers informed that their identity would be revealed) or blinded peer review (i.e., peer reviewers blinded to author names and affiliation); and interventions to increase the speed of the peer review process (10). The authors refrain from providing recommendations regarding the wider implementation of the identified interventions due to their low methodological quality. Other researchers have argued that most of the approaches outlined above fail to compensate for potential biases, and point out that any success so far has been limited (11).

For peer review only - http://bmjopen.bmj.com/site/about/guidelines.xhtml

Researchers have argued that limited success of interventions might be due to the underlying nature of peer review, which has been described as an intellectual process that encompasses objective and subjective elements (12). Editors and peer reviewers bring a diverse mix of skills, preferences, and intellectual idiosyncrasies to the task (13). At times, these may result in subjective judgements of manuscript quality. Peer review has also been described as an "inherently human phenomenon" that is underpinned by social dimensions (14,15). A qualitative study of the social and subjective dimensions of manuscript peer review in biomedical publishing concluded that biomedical manuscript review *"is a highly social and subjective process driven by communal as well as scientific goals, and influenced by reviewers' and editors' sense of their own authority, power, and moral responsibility, as well as by unavoidable prejudice and intuition."* (16).

Our broader research framework aims to generate an understanding of the communication practices within the editorial and manuscript peer review process in biomedical research. Disentangling current communication practices for a range of biomedical journals, and outlining the specific roles and tasks of the main actors might be a first step towards establishing the design of interventions that counterbalance social influences on the peer review process. In this study, we aim to identify and characterize the roles and tasks of the different actors in the process of peer review from the perspective of journal editors.

Our specific objectives are:

1) To examine biomedical editors' experiences of their interactions with peer reviewers and authors
2) To characterize journal editors' perspectives regarding the roles and tasks of peer reviewers
3) To explore journal editors' experiences of communication practices within the manuscript review process and their views regarding such practices' potential impact on research output, namely the fate of individual manuscripts.

Methods and analysis

Qualitative approach and research paradigm

Given its underlying social and subjective dimensions (16,17), the need for more qualitative research into the peer review process within the biomedical field has been recognized for some time (1). However, to date, most such research has been overwhelmingly quantitative in nature (18).

Drawing on a pragmatist epistemological position that the aim of inquiry cannot be independent from human experience (19), we considered a qualitative approach to be best suited to answer our research question. The expectations, understandings, perceptions and thoughts of journal editors are largely intangible aspects that cannot be unpacked using predefined categories or viewed independently from the purposes of the peer review process itself.

For peer review only - http://bmjopen.bmj.com/site/about/guidelines.xhtml

The use of qualitative interviews will enable participants to speak freely and at length about such aspects, thus providing rich data embedded in personal experiences and practices.

Data will be analysed using thematic analysis as described by Braun and Clarke primarily because of the method's flexibility and epistemological assumptions, that are compatible with a pragmatist approach (20).

Study participants will be offered the possibility of conducting the interview either face-to-face or by phone/conferencing system, according to personal preference and availability. This will also enable the interviewing of people in geographically distant locations.

Study sample and recruitment

We will use maximum variation purposive sampling to recruit a heterogeneous study sample, allowing us to select journal editors with different characteristics that we anticipate may influence their perspectives. This sampling method enables conceptual exploration using the characteristics of individuals and journals as the basis of selection in order to reflect the diversity and breadth of the sample population, rather than achieving population representativeness (21).

Participants will be recruited through different sources. The study recruitment pathway is shown in Table 1.

Table 1. Study recruitment pathways

**Source of participants**	**Sampling**
Existing professional networks	Purposive/Snowballing
Two research publishers	Purposive/Snowballing
International Congress on Peer Review and Scientific Publication	Purposive/Snowballing
Editor Associations	Purposive/Snowballing

Initially, interviewees will be drawn from a professional network of contacts (members of the Methods in Research on Research (MiRoR) project (22)) who are journal editors. They will be interviewed for piloting purposes and requested to recommend additional journal editors whom the lead investigator can interview.

The research publishers BMC (part of Springer Nature) and BMJ are partners of the MiRoR project.

This partnership will be used to recruit interviewees. Editors-in chief operating within the BMJ Publishing Group will be contacted by the lead researcher (KG) via email, provided with study details, and asked to either participate themselves or recommend suitable journal editors who can be contacted instead. One follow-up email will be sent after two weeks to non-respondents.

Due to a different standard operating procedure a different recruitment strategy will be employed at BMC. The publishers' communication manager will communicate with all editors via internal

For peer review only - http://bmjopen.bmj.com/site/about/guidelines.xhtml

mail, introduce the lead researcher and the research, and encourage them to respond if interested in participating.

Concurrently, the conference participation lists from the Eight International Congress on Peer Review and Scientific Publication (23) will be reviewed and potential interviewees will be contacted via email by the lead researcher. One follow-up email will be sent to non-respondents after two weeks.

In addition, Editor Associations (Table 2) may be contacted to recruit further study participants in order to obtain maximum variability of editors' characteristics.

Table 2 – List of Editors Associations

Asia pacific association of medical journal editors (APAME)
Council of Science Editors (CSE)
European Association of Science Editors (EASE)
Eastern Mediterranean Association of Medical Editors (EMAME)
Forum for African Medical Editors (FAME)
International Committee of Medical Journal Editors (ICMJE)
Japanese Association of Medical Journal Editors (JAMJE)
Korean Association of Medical Journal Editors (KAMJE)
Philippine Association of Medical Journal Editors (PAMJE)
World Association of Medical Editors (WAME)

Following the maximum variation sampling strategy, journal editors who agree to be interviewed will be categorized using the characteristics presented in Table 3, some of which have been shown to influence the peer review process (e.g. gender) (24).
This step will help to determine the characteristics that are under-represented, and inform the sampling strategy for identification of further participants in such a way as to maximize the diversity of interviewees.

Table 3 – Sample characteristics

Criteria	Characteristics
Demographic characteristics	• Gender<br>• Editorial experience<br>• Editors' geographic location
Journal characteristics	• Journal specialty (e.g. Clinical, Public

For peer review only - http://bmjopen.bmj.com/site/about/guidelines.xhtml

	Health)<br>• Impact factor (journals with or without impact factor)<br>• Peer review practices (closed peer review, open peer review, post-publication peer review)<br>• Publisher (medical publishing companies, independent publisher/university)<br>• Open access, paywall<br>• Size (editorial team)

Lastly, the journal editor identification process will be supplemented through snowball sampling (25). At the end of each interview, interviewees will be asked to recommend other editors whose experiences might be relevant to the study and who would potentially be interested in contributing to this study. These steps are expected to lead to recommendations that optimize sample variation.

Saturation

Saturation is a core guiding principle to determine sample sizes in qualitative research, yet few qualitative studies report in detail on the parameters that influenced saturation in their studies (26). In this study we will adopt the seven parameters outlined by Hennink et al. that influence saturation (27) to establish our sample size determinants and demonstrate the grounds upon which saturation will be assessed and achieved, thereby justifying the final sample size. The parameters of saturation and sample size for our study are outlined in Table 4.

The first parameter is the *purpose* of the study, which in this case is to capture themes from the data by using the thematic analysis method. The second parameter is *population,* which should ideally be as heterogeneous as possible. This parameter will be satisfied by interviewing journal editors with different characteristics (i.e. demographic characteristics, journal specialty and journal characteristics). Our *data collection strategy* will be iterative, involving continual data collection until saturation is reached. We aim to collect *thick data*: developing conceptual codes that capture subtle issues. We will develop our *codebook* so that it includes a broad range of codes, including explicit, subtle, and conceptual codes. Lastly, the *saturation goal and focus* of our study is to achieve full understanding of the issues and meanings that emerge from the data. Thus, we aim to reach meaning saturation rather than code saturation (27). Although the process of reaching saturation cannot be quantified in advance and involves an iterative approach until saturation is obtained, we used a recently developed quantitative method to offer an initial estimate of expected number of participants in our study. Following the approach suggested by Fugard and Potts (28) of estimating sample size required to achieve code saturation for studies that use thematic analysis, we calculated that we would need a sample size of at least 38 participants to detect, with 90% power, two instances of a theme with 10% prevalence. Appendix 1 shows the details of the

For peer review only - http://bmjopen.bmj.com/site/about/guidelines.xhtml

calculation. This is in line with our previously hypothesized number of participants. Therefore, whilst our core approach to data collection strategy will be iterative, involving continual data collection until saturation is reached, we anticipate around 40 participants to be sufficient to provide us with meaningful information to answer our research questions, in line with similar studies (16).

Table 4 – Parameters of saturation and sample size

Parameters	Sample size determinants of our study	Our study
Purpose	Capture themes	The thematic analysis method will be used to identify themes and patterns of meanings across the dataset in relation to the research question
Population	Heterogeneous	Journal editors with different characteristics (i.e. demographic characteristics, journal discipline and characteristics)
Sampling strategy	Iterative sample	Purposive sampling using established networks; enlarged through snowballing
Data quality	Thick data	Experiences and opinions will be captured
Type of codes	Conceptual codes	Explicit and subtle
Codebook	Emerging codebook	Inductive coding derived from data content
Saturation goal and focus	Code meaning	Referring to saturation as the point where the issues are fully understood and when no further dimensions, nuances, and insights can be found

Inclusion criteria and recruitment process

Study participants will consist of journal editors of biomedical journals, referring to individuals who are currently involved in the communication process between authors and peer reviewers and therefore in a position to decide about the fate of manuscripts; and/or contribute to the determination of journal content and policy. Journal editors will be contacted between October 2017 and February 2018. They will be sent an invitation email and information sheet by the lead author (KG), followed by a phone call to determine if they are interested in participating in the study. Written informed consent will be obtained from all participants prior to conducting the interviews.

Interview guide

A preliminary topic guide for the semi-structured interviews (see Table 5) has been developed, informed by the outcomes of a previously conducted scoping review of the literature (29). The

For peer review only - http://bmjopen.bmj.com/site/about/guidelines.xhtml

topic guide was piloted on four editors to assess usefulness and meaningfulness of the questions, the ease of administration, language and length, and to refine the topic guide. It is likely that the topic guide will be refined further after conducting more interviews.

Table 5 – Draft topic guide for semi-structured interviews

**Key area of investigation**	**Topics**	**Questions and prompts**
Background information	- Explore personal background<br>- Level of experience<br>- Own roles and tasks as an editor	• Tell me about your journal and the job you have.<br>• How long have you been in this position?<br>• What are your current responsibilities?<br>• Did you hold any other positions in the same field before your current position? If yes, what were your responsibilities then?
Journal set-up	- Explore journal set-up	• Tell me about your journal - how does it work?<br>• How does the peer review process work in your journal?<br>• What do you do within the process?
Opinion on peer reviewers role and tasks	- Roles and tasks of peer reviewers<br>- Expectations	• What do you expect from peer reviewers in terms of their roles and tasks?<br>• How do you let your reviewers know what you expect from them?<br>**Prompt** on whatever has not been mentioned:<br>• Attitudes and beliefs (about role and tasks)<br>• Organisational expectations (about role and tasks)<br><br>• Can you tell me about a specific situation when you were not satisfied with a review or with a peer reviewer?<br>• What did you do in that a situation?<br>**Prompt**: looks for factors other than being late with a review, or not doing a review once you they have accepted it<br><br>• Can you tell me about a situation when you were exceptionally satisfied with a review or with a peer reviewer?<br>• Were there situations (in regards to the roles and task of reviewers) when you disagreed with the other editors you work with? What about? What happened?<br>• What about other journals, do roles and tasks differ among journals in your field?<br>**Prompt**: If yes (i.e. differences exist), then:<br>• How does this affect the process?<br>• How does it affect your communication?<br>• How do you negotiate those differences? Does it

For peer review only - http://bmjopen.bmj.com/site/about/guidelines.xhtml

		matter?
Communication between editors, peer reviewers and authors	- Communication between the three parties<br>- Potential conflicts<br>- Power	• Can you describe your experience of the communication process between editors, authors and peer reviews?<br>• How do you communicate with authors and peer reviewers?<br>• Can you give me some specific examples of situations where this communication is challenging?<br>**Prompt**:<br>What are potential conflicts?<br>When do disagreements arise?
Conclusion	- Snowballing<br>- Documents<br>- Final comments	• Is there anybody else whom you think I should speak to?<br>• Any articles/documents I can access/should look at?<br>• Any final comments? Is there anything else that you think is important to mention?

Data collection and recording

All interviews will be conducted by the principal researcher (KG) either face-to-face or by phone or online call (e.g. Skype or conferencing system), according to the circumstances and preferences of the interviewees.

With the permission of the participants, interviews will be audio-recorded and notes will be taken. Interviewees will be asked if they could be contacted again if further clarification is needed.

Based on the pilot interviews it is anticipated that interviews will take around 30 minutes to complete.

Data analysis

Data will be analysed using Braun and Clarke's (20) 6-phase method for thematic analysis (TA). This analytic framework assumes that truth can be accessed through language, but that accounts and experiences are socially mediated (30). It is not bound to any pre-existing theoretical framework, therefore it offers relative theoretical independence and compatibility with various approaches which is compatible with pragmatist position that we subscribe to (31). TA has also been described as a more accessible form of analysis compared to other approaches (e.g. grounded theory) that requires less detailed theoretical and technical knowledge, and is therefore particularly suitable for the lead researcher (KG) of this study who is at an early stage of her qualitative research experience (20).

The principal researcher will conduct all interviews, which will be transcribed verbatim.

An Excel spreadsheet will be used to log all details related to data collection (e.g. interview schedule, field notes) processing (e.g. transcription, coding and analysis) and progress throughout.

Data analysis will take place concurrently with data collection in an iterative cycle. This serves two purposes; firstly, it will help to further refine the topic guide, and allow the interviewer (KG) to reflect with the senior investigator (DH) on her own interviewing technique and style for subsequent interviews. Secondly, it will help the researchers to determine when saturation occurs.

For peer review only - http://bmjopen.bmj.com/site/about/guidelines.xhtml

The process will begin with initial reflections after each interview. These will be recorded (in the Excel sheet) as field notes. This diary will be kept during data collection and data analysis to capture impressions from interviews (such as initial themes) and highlight the decision-making process leading to the development of initial and final themes.

The step-by-step approach by Nowell at al. that provides a detailed description and pragmatic approach of how to conduct a trustworthy TA will be followed (32).

The first phase will start by familiarizing with the data - re-reading each transcript at least twice and noting down initial ideas.

In the second phase initial codes will be generated across the entire dataset using the inductive approach, whereby codes are developed line-by-line from the interview content, focusing on the identification of both semantic (i.e. reflecting the explicit content) and latent (i.e. reflect the implicit content) features (20). All coding will be performed by both researchers KG and DH to increase credibility of the analysis. The qualitative data analysis software NVivo v.11 will be used to aid data management and analysis (i.e. indexing of coding and transcripts). In order to ensure consistency a code manual will be developed prior to the in-depth analysis.

In the third phase the codes will be clustered into potential themes to give an indication of their prevalence for the assessment of (code or meaning) saturation, and into a preliminary thematic map displaying the main themes.

The fourth phase will consist of reviewing themes and will be divided into two stages; the reviewing and refining of the data at the level of the coded data extracts, and subsequently at the level of the entire data set. These two stages will lead to the generation of a thematic map of the analysis.

The aim of the next phase will be to definitively define the scope and content of each relevant theme and precisely name them. This will involve debriefing between the study team. Debriefing with an outside expert (on peer review in biomedical journals) as suggested by King (33) will be conducted to ensure that themes are sufficiently clear to someone outside of the immediate research team.

After the establishment of the final themes, the last phase will consist of writing up the study findings as a journal article. Direct quotes will be used to illustrate specific points of interpretation and the extraction of themes. All themes and subthemes will be presented in the result section and discussed in the light of existing literature.

Securing study quality

In order to establish trustworthiness and credibility of data analysis the practical procedures outlined by Nowell at al. will be followed at each stage of analysis (32).

Additionally, respondent validation will be sought by inviting interviewees to comment on the interpretation of their interview (i.e. final themes and coding) (34).

For peer review only - http://bmjopen.bmj.com/site/about/guidelines.xhtml

We will use the consolidated criteria for reporting qualitative research (COREQ) to provide detailed reporting of methods used (35).

Ethical considerations

Interviewees will receive an information sheet about the research and a consent form before the interview. The information letter includes details on the maintenance of anonymity and confidentiality throughout the research process. Prior to the interview, information from the information sheet and consent form will be reiterated verbally, and interviewees will be asked to consent to participation and recording of their interviews. Participants will be able to choose not to be directly quoted in any publications resulting from the study.

Potential use of the study

This research has multiple potential uses. As a standalone research piece it will generate context-based information from journal editors' perspectives that will help to provide insight into the communication patterns within biomedical journals, including differences and similarities across biomedical journals. It is also embedded within a larger project that will inform the development of a taxonomy of peer reviewers' roles and tasks, potentially leading to the establishment of a set of core competencies for peer reviewers of biomedical journals.

The study findings can further be used to inform biomedical journal policies and develop training courses for peer reviewers and journal editors.

Ethical approval This project has been evaluated and approved by the University of Split, Medical School Ethics Committee (2181-198-03-04-17-0029) in May 2017.

Acknowledgements

The authors would like to thank Professor Erik Cobo for providing advice during the writing of this protocol and Dr Sara Schroter (BMJ) and Dr Elizabeth Moylan (BMC) for providing guidance and help on the recruitment strategy of interviewees.

Contributors All authors have made substantive intellectual contributions to the development of this protocol. KG conceptualized the study approach and led the writing of the manuscript. DH led the supervision of the manuscript preparation. DH was involved in developing the study questions and design and provided detailed comments on earlier drafts.

Funding This project was supported by the European Union's Horizon 2020 research and innovation programme under the Marie Sklodowska-Curie grant agreement No 676207. The funders had no role in the study design, data collection and analysis, decision to publish, or preparation of the manuscripts.

Competing interests At the time of the submission of this protocol KG conducted a secondment at the BMJ.

For peer review only - http://bmjopen.bmj.com/site/about/guidelines.xhtml

References

1. Overbeke J, Wager E. 3: The state of evidence: what we know and what we don't know about journal peer review. JAMA. 1994;272:79–174.

2. Galipeau J, Barbour V, Baskin P, Bell-Syer S, Cobey K, Cumpston M, et al. A scoping review of competencies for scientific editors of biomedical journals. BMC Med. 2016 Feb 2;14:1–17.

3. Moher D, Galipeau J, Alam S, Barbour V, Bartolomeos K, Baskin P, et al. Core competencies for scientific editors of biomedical journals: consensus statement. BMC Med. 2017 Sep 11;15(1):167.

4. Smith R. Peer review: a flawed process at the heart of science and journals. J R Soc Med [Internet]. 2006;99. Available from: http://dx.doi.org/10.1258/jrsm.99.4.178

5. Rennie D. Make peer review scientific. Nature. 2016;535(7610):31–3.

6. Silva PUKD, Vance CK. Preserving the Quality of Scientific Research: Peer Review of Research Articles. In: Scientific Scholarly Communication [Internet]. Springer International Publishing; 2017 [cited 2017 Apr 18]. p. 73–99. (Fascinating Life Sciences). Available from: http://link.springer.com/chapter/10.1007/978-3-319-50627-2_6

7. Lee CJ, Sugimoto CR, Zhang G, Cronin B. Bias in peer review. J Am Soc Inf Sci Technol. 2013 Jan 1;64(1):2–17.

8. Jefferson T, Rudin M, Brodney Folse S. Editorial peer review for improving the quality of reports of biomedical studies. Cochrane Database Syst Rev. 2007;2.

9. Altman DG, Schulz KF, Moher D. The revised CONSORT statement for reporting randomized trials: explanation and elaboration. Ann Intern Med [Internet]. 2001;134. Available from: http://dx.doi.org/10.7326/0003-4819-134-8-200104170-00012

10. Bruce R, Chauvin A, Trinquart L, Ravaud P, Boutron I. Impact of interventions to improve the quality of peer review of biomedical journals: a systematic review and meta-analysis. BMC Med. 2016;14(1):85.

11. Hojat M, Gonnella JS, Caelleigh AS. Impartial judgment by the "gatekeepers" of science: fallibility and accountability in the peer review process. Adv Health Sci Educ Theory Pract. 2003;8(1):75–96.

12. Kassirer JP, Campion EW. Peer review: crude and understudied, but indispensable. Jama. 1994;272(2):96–7.

13. King CR, McGuire DB, Longman AJ, Carroll-Johnson RM. Peer review, authorship, ethics, and conflict of interest. Image J Nurs Sch. 1997 May;29(2):163–7.

14. Horn SA. The Social and Psychological Costs of Peer Review: Stress and Coping With Manuscript Rejection. J Manag Inq. 2016 Jan 1;25(1):11–26.

For peer review only

For peer review only - http://bmjopen.bmj.com/site/about/guidelines.xhtml

15. Bedeian AG. Peer Review and the Social Construction of Knowledge in the Management Discipline. Acad Manag Learn Educ. 2004 Jun 1;3(2):198–216.

16. Lipworth WL, Kerridge IH, Carter SM, Little M. Journal peer review in context: A qualitative study of the social and subjective dimensions of manuscript review in biomedical publishing. Soc Sci Med. 2011 Apr;72(7):1056–63.

17. Lipworth W, Kerridge I. Shifting Power Relations and the Ethics of Journal Peer Review. Soc Epistemol. 2011 Jan 1;25(1):97–121.

18. Shashok K. Standardization vs Diversity: How Can We Push Peer Review Research Forward? Medscape Gen Med. 2005 Feb 16;7(1):11.

19. Yardley L, Bishop F. Mixing Qualitative and Quantitative Methods: A Pragmatic Approach. In: Willig C, Stainton-Rogers W, editors. Qualitative Research in Psychology. Thousand Oaks (CA): SAGE Publication Ltd.; p. 352–69.

20. Braun V, Clarke V. Using thematic analysis in psychology. Qual Res Psychol. 2006;3(2):77–101.

21. Patton M. Qualitative Research and Evaluation Methods , 209-339. 2002;

22. Projet MiRoR | An innovative and ambitious joint doctoral training programme [Internet]. [cited 2017 May 2]. Available from: http://miror-ejd.eu/

23. Rennie D, Flanagin A, Godlee F, Bloom T. The Eighth International Congress on Peer Review and Biomedical Publication: A Call for Research. JAMA. 2015 May 26;313(20):2031–2.

24. Fox CW, Burns CS, Meyer JA. Editor and reviewer gender influence the peer review process but not peer review outcomes at an ecology journal. Funct Ecol. 2016;30(1):140–53.

25. Morgan DL. Sample size. Sage Encycl Qual Res Methods. 2008;798.

26. Marshall B, Cardon P, Poddar A, Fontenot R. Does Sample Size Matter in Qualitative Research?: A Review of Qualitative Interviews in is Research. J Comput Inf Syst. 2013 Sep 1;54(1):11–22.

27. Hennink MM, Kaiser BN, Marconi VC. Code Saturation Versus Meaning Saturation: How Many Interviews Are Enough? Qual Health Res. 2016;1049732316665344.

28. Fugard AJB, Potts HWW. Supporting thinking on sample sizes for thematic analyses: a quantitative tool. Int J Soc Res Methodol. 2015 Nov 2;18(6):669–84.

29. Glonti K, Cauchi D, Cobo E, Boutron I, Moher D, Hren D. A scoping review protocol on the roles and tasks of peer reviewers in the manuscript review process in biomedical journals. BMJ Open. 2017 Oct 1;7(10):e017468.

For peer review only - http://bmjopen.bmj.com/site/about/guidelines.xhtml

30. Madill A, Jordan A, Shirley C. Objectivity and reliability in qualitative analysis: realist, contextualist and radical constructionist epistemologies. Br J Psychol Lond Engl 1953. 2000 Feb;91 (Pt 1):1–20.

31. Creswell JW, Poth CN. Qualitative inquiry and research design: Choosing among five approaches. Sage publications; 2017.

32. Thematic AnalysisInternational Journal of Qualitative Methods - Lorelli S. Nowell, Jill M. Norris, Deborah E. White, Nancy J. Moules, 2017 [Internet]. [cited 2017 Oct 31]. Available from: http://journals.sagepub.com.liverpool.idm.oclc.org/doi/full/10.1177/1609406917733847

33. King N. Using templates in the thematic analysis of text. Essent Guide Qual Methods Organ Res. 2004;2:256–70.

34. Long T, Johnson M. Rigour, reliability and validity in qualitative research. Clin Eff Nurs. 2000;4(1):30–7.

35. Tong A, Sainsbury P, Craig J. Consolidated criteria for reporting qualitative research (COREQ): a 32-item checklist for interviews and focus groups. Int J Qual Health Care. 2007 Dec 1;19(6):349–57.

For peer review only

For peer review only - http://bmjopen.bmj.com/site/about/guidelines.xhtml

Appendix 1. Sample size calculation

```
sampSizeForQual = function(Power, ThemePrevalence, NumInstances) { qnbinom(Power, size =
NumInstances, prob = ThemePrevalence) + NumInstances

}

sampSizeForQual(0.9, 0.1, 2)

[1] 38
```

Code run via R-Fiddle (httm://www.r-fiddle.org)

For peer review only

BMJ Open

The editors' perspectives on the peer review process in biomedical journals: Protocol for a qualitative study

Journal:	*BMJ Open*
Manuscript ID	bmjopen-2017-020568.R1
Article Type:	Protocol
Date Submitted by the Author:	15-May-2018
Complete List of Authors:	Glonti, Ketevan; Sveuciliste u Splitu, School of Humanities and Social Sciences; Paris Descartes University, Sorbonne Paris Cité, INSERM, U1153 Epidemiology and Biostatistics Sorbonne Paris Cité Research Center (CRESS), Methods of therapeutic evaluation of chronic diseases Team (METHODS)<br>Hren, Darko; University of Split, School of Humanities and Social Sciences
**Primary Subject Heading**:	Medical publishing and peer review
Secondary Subject Heading:	Qualitative research
Keywords:	peer review, QUALITATIVE RESEARCH, scientific publishing

SCHOLARONE™ Manuscripts

The editors' perspectives on the peer review process in biomedical journals: Protocol for a qualitative study

Authors: Ketevan Glonti[1, 2], Darko Hren[1]

[1] University of Split, School of Humanities and Social Sciences, Split, Croatia. kglonti@unist.hr, dhren@unist.hr

[2] INSERM, U1153 Epidemiology and Biostatistics Sorbonne Paris Cité Research Center (CRESS), Methods of therapeutic evaluation of chronic diseases Team (METHODS), Paris, F-75014 France; Paris Descartes University, Sorbonne Paris Cité, France.

Corresponding author: Ketevan Glonti, Poljička 35, 21000 Split, Croatia, kglonti@unist.hr

Tel: +385 976324635

For peer review only

Abstract

Introduction: Despite dealing with scientific output and potentially having an impact on the quality of research published, the manuscript peer review process itself has at times been criticised for being 'unscientific'. Research indicates that there are social and subjective dimensions of the peer review process that contribute to this perception, including how key stakeholders - namely authors, editors and peer reviewers - communicate. In particular, it has been suggested that the expected roles and tasks of stakeholders need to be more clearly defined and communicated if the manuscript review process is to be improved. Disentangling current communication practices, and outlining the specific roles and tasks of the main actors, might be a first step towards establishing the design of interventions that counterbalance social influences on the peer review process.

The purpose of this article is to present a methodological design for a qualitative study exploring the communication practices within the manuscript review process of biomedical journals from the journal editors' point of view.

Methods and analysis: Semi-structured interviews will be carried out with editors of biomedical journals between October 2017 and February 2018. A heterogeneous sample of participants representing a wide range of biomedical journals will be sought through purposive maximum variation sampling, drawing from a professional network of contacts, publishers, conference participants, editors associations and snowballing.

Interviews will be thematically analysed following the method outlined by Braun and Clarke. The qualitative data analysis software NVivo v11 will be used to aid data management and analysis.

Ethics and dissemination: This research project was evaluated and approved by the University of Split, Medical School Ethics Committee (2181-198-03-04-17-0029) in May 2017. Findings will be disseminated through a publication in a peer-reviewed journal and presentations during conferences.

For peer review only - http://bmjopen.bmj.com/site/about/guidelines.xhtml

Keywords: Peer review, Biomedical publishing, Scientific journal publishing, Qualitative research

Strengths and limitations of this study

- Qualitative analysis of interview data from a wide range of editors of biomedical journals will allow an in-depth understanding of the communication practices prevailing within biomedical journals.
- Quality assurance will be employed throughout data collection and analysis to ensure traceability and verification.
- Journal editors of a selection of biomedical journals will be interviewed; therefore, research findings cannot directly be extrapolated to all biomedical journals and other scientific fields.

For peer review only

Introduction

Most journals in the biomedical field implement a pre-publication process which primarily involves the interaction of three key stakeholders around an academic research manuscript: journal editors, peer reviewers and authors. This process, typically referred to as 'peer review', is strongly embedded in the field of biomedical publishing and in its broadest sense refers to the evaluation of manuscripts submitted for publication by researchers from the same or related areas of expertise. Thus far, there is no universal definition of 'peer review', and its specific objectives are not clearly defined (1). Concurrently, the roles, tasks and core competencies expected of peer reviewers and editors have not been formally established and both groups operate largely without formal training (2,3). A study that aimed to identify all tasks that are expected of peer reviewers by journal editors when evaluating a manuscript reporting a randomized controlled trial (RCT) found that the most important tasks in peer review, as perceived by peer reviewers evaluating RCTs, were not congruent with the tasks most often requested by journal editors in their guidelines to reviewers (4) These differences illustrate the need to clarify the roles and tasks of peer reviewers.

The peer review process has at times been criticised for being 'unscientific' (5,6). Despite dealing with scientific output that potentially leads to changes in clinical practice, the process itself is not without potential biases - including prestige or association bias, gender bias, confirmation bias, conservatism, bias against interdisciplinary research, publication bias, language bias, and conflict of interest (7,8).

In light of this criticism, there have been several attempts to improve the peer review process and the quality of peer reviewer reports in the biomedical field (9). A recent systematic review evaluating the impact of interventions to improve the quality of peer review for biomedical publications (10). identified 25 strategies that have been implemented, including training interventions; use of checklists (such as Consort (11)); addition of specific experts (i.e. statistical peer reviewers); the introduction of open peer review (i.e., peer reviewers informed that their identity would be revealed) or blinded peer review (i.e., peer reviewers blinded to author names and affiliation); and interventions to increase the speed of the peer review process. The authors of the systematic review refrain from providing recommendations regarding the wider

For peer review only - http://bmjopen.bmj.com/site/about/guidelines.xhtml

implementation of the identified interventions due to concerns about their methodological quality, small sample size and applicability. Other researchers have argued that most of the approaches outlined above fail to compensate for potential biases, and point out that any success so far has been limited (12).
Researchers have argued that limited success of interventions might be due to the underlying nature of peer review, which has been described as an intellectual process that encompasses objective and subjective elements (13). Editors and peer reviewers bring a diverse mix of skills, preferences, and intellectual idiosyncrasies to the task (14). At times, these may result in subjective judgements of manuscript quality. Peer review has also been described as an "inherently human phenomenon" that is underpinned by social dimensions (15,16). A qualitative study of the social and subjective dimensions of manuscript peer review in biomedical publishing concluded that biomedical manuscript review *"is a highly social and subjective process driven by communal as well as scientific goals, and influenced by reviewers' and editors' sense of their own authority, power, and moral responsibility, as well as by unavoidable prejudice and intuition."* (17).

Our broader research framework aims to generate an understanding of the communication practices within the editorial and manuscript peer review process in biomedical research. Disentangling current communication practices for a range of biomedical journals, and outlining the specific roles and tasks of the main actors might be a first step towards establishing the design of interventions that counterbalance social influences on the peer review process. In this study, we aim to identify and characterize the roles and tasks of the different actors in the process of peer review from the perspective of journal editors.

Our specific objectives are:

1) To examine biomedical editors' experiences of their interactions with peer reviewers and authors
2) To characterize journal editors' perspectives, expectations, understandings and perceptions regarding the roles and tasks of peer reviewers

Methods and analysis

Qualitative approach and research paradigm

Given its underlying social and subjective dimensions (17,18), the need for more qualitative research into the peer review process within the biomedical field has been recognized for some time (1). However, to date, most such research has been overwhelmingly quantitative in nature (19).

Drawing on a pragmatist epistemological position that the aim of inquiry cannot be independent from human experience (20), we considered a qualitative approach to be best suited to answer our research question. The expectations, understandings, perceptions and thoughts of journal editors

For peer review only - http://bmjopen.bmj.com/site/about/guidelines.xhtml

are largely intangible aspects that cannot be unpacked using predefined categories or viewed independently from the purposes of the peer review process itself.
The use of qualitative interviews will enable participants to speak freely and at length about such aspects, thus providing rich data embedded in personal experiences and practices.
Data will be analysed using thematic analysis as described by Braun and Clarke primarily because of the method's flexibility and epistemological assumptions, that are compatible with a pragmatist approach (21).
Study participants will be offered the possibility of conducting the interview either face-to-face or by phone/conferencing system, according to personal preference and availability. This will also enable the interviewing of people in geographically distant locations.

Study sample and recruitment

We will use maximum variation purposive sampling to recruit a heterogeneous study sample of biomedical journal editors, allowing us to select editors with different characteristics that we anticipate may influence their perspectives. This sampling method enables conceptual exploration using the characteristics of individuals and journals as the basis of selection in order to reflect the diversity and breadth of the sample population, rather than achieving population representativeness (22).
Participants will be recruited through different sources. The study recruitment pathway is shown in Table 1.

Table 1. Study recruitment pathways

**Source of participants**	**Sampling**
Existing professional networks	Purposive/Snowballing
Two research publishers	Purposive/Snowballing
International Congress on Peer Review and Scientific Publication	Purposive/Snowballing
Editor Associations	Purposive/Snowballing

Initially, interviewees will be drawn from a professional network of contacts (members of the Methods in Research on Research (MiRoR) project (23)) who are journal editors. Four editors will be interviewed for piloting purposes and requested to recommend additional journal editors whom the lead investigator can interview.

The research publishers BMC (part of Springer Nature) and BMJ are partners of the MiRoR project.
This partnership will be used to recruit interviewees. Editors-in chief operating within the BMJ Publishing Group will be contacted by the lead researcher (KG) via email, provided with study details, and asked to either participate themselves or recommend suitable journal editors who can be contacted instead. One follow-up email will be sent after two weeks to non-respondents.

For peer review only - http://bmjopen.bmj.com/site/about/guidelines.xhtml

Due to a different standard operating procedure a different recruitment strategy will be employed at BMC. The publishers' communication manager will communicate with all editors via internal mail, introduce the lead researcher and the research, and encourage them to respond if interested in participating.

Concurrently, the conference participation lists from the Eighth International Congress on Peer Review and Scientific Publication (24) will be reviewed and potential interviewees will be contacted via email by the lead researcher. One follow-up email will be sent to non-respondents after two weeks.

In addition, Editor Associations (Table 2) may be contacted to recruit further study participants in order to obtain maximum variability of editors' characteristics.

Table 2 – List of Editors Associations

Asia pacific association of medical journal editors (APAME)
Council of Science Editors (CSE)
European Association of Science Editors (EASE)
Eastern Mediterranean Association of Medical Editors (EMAME)
Forum for African Medical Editors (FAME)
International Committee of Medical Journal Editors (ICMJE)
Japanese Association of Medical Journal Editors (JAMJE)
Korean Association of Medical Journal Editors (KAMJE)
Philippine Association of Medical Journal Editors (PAMJE)
World Association of Medical Editors (WAME)

Following the maximum variation sampling strategy, journal editors who agree to be interviewed will be categorized using the characteristics presented in Table 3, some of which have been shown to influence the peer review process (e.g. gender) (25).
This step will help to determine the characteristics that are under-represented, and inform the sampling strategy for identification of further participants in such a way as to maximize the diversity of interviewees.

Table 3 – Sample characteristics

**Criteria**	**Characteristics**
Demographic characteristics	• Gender<br>• Editorial experience

For peer review only - http://bmjopen.bmj.com/site/about/guidelines.xhtml

	• Editors' geographic location
Journal characteristics	• Journal specialty (e.g. Clinical, Public Health)<br>• Impact factor (journals with or without impact factor)<br>• Peer review practices (closed peer review, open peer review, post-publication peer review)<br>• Publisher (medical publishing companies, independent publisher/university)<br>• Open access, paywall<br>• Size (editorial team)

Lastly, the journal editor identification process will be supplemented through snowball sampling (26). At the end of each interview, interviewees will be asked to recommend other editors whose experiences might be relevant to the study and who would potentially be interested in contributing to this study. These steps are expected to lead to recommendations that optimize sample variation.

Saturation

Saturation is a core guiding principle to determine sample sizes in qualitative research, yet few qualitative studies report in detail on the parameters that influenced saturation in their studies (27). In this study we will adopt the seven parameters outlined by Hennink et al. that influence saturation (28) to establish our sample size determinants and demonstrate the grounds upon which saturation will be assessed and achieved, thereby justifying the final sample size. The parameters of saturation and sample size for our study are outlined in Table 4. According to Hennink et al. the sample size is determined by the combined influence of all parameters rather than any single parameter alone. In our case some parameters indicate a smaller sample for saturation and others suggest a larger sample, suggesting the need for an intermediate sample size.

The first parameter is the *purpose* of the study, which in this case is to capture themes from the data using the thematic analysis method. The second parameter is *population.* For the purposes of our study we want do grasp as wide variety of biomedical editors as possible and will thus obtain a heterogeneous sample. This parameter will be satisfied by interviewing journal editors with different characteristics (i.e. demographic characteristics, journal specialty and journal characteristics). Our *data collection strategy* will be iterative, involving continual data collection until saturation is reached. We aim to collect *thick data* in order to provide deep and rich insights and capture explicit and concrete codes as well as conceptual codes that capture subtle issues. Our *codebook will be emerging including* a broad range of codes, including explicit, subtle, and conceptual codes.

For peer review only - http://bmjopen.bmj.com/site/about/guidelines.xhtml

Lastly, the *saturation goal and focus* of our study is to achieve data saturation i.e. the point where no new issues or themes are identified from the data (28).

Although the process of reaching saturation cannot be quantified in advance and involves an iterative approach until saturation is obtained, we used a recently developed quantitative method to offer an initial estimate of expected number of participants in our study. Following the approach suggested by Fugard and Potts (29) of estimating sample size required to achieve code saturation for studies that use thematic analysis, we calculated that we would need a sample size of at least 38 participants to detect, with 90% power, two instances of a theme with 10% prevalence. Appendix 1 shows the details of the calculation. This is in line with our previously hypothesized number of participants. Therefore, whilst our core approach to data collection strategy will be iterative, involving continual data collection until saturation is reached, we anticipate around 40 participants to be sufficient to provide us with meaningful information to answer our research questions, in line with similar studies (17).

Table 4 – Parameters of saturation and determinants of sample size for our study

**Parameters**	**Sample size determinant for each parameter**	**Determinant definition**
Purpose	Capture themes	The thematic analysis method will be used to identify themes and patterns of meanings across the dataset in relation to the research question
Population	Heterogeneous	Journal editors with different characteristics (i.e. demographic characteristics, journal discipline and characteristics)
Sampling strategy	Iterative sample	Purposive sampling using established networks; enlarged through snowballing
Data quality	Thick data	Experiences and opinions will be captured with the aim to provide deep and rich insights
Type of codes	Conceptual codes	Explicit and subtle
Codebook	Emerging codebook	Inductive coding derived from data content including broad range of codes
Saturation goal and focus	Data saturation	Referring to saturation as the point where no new issues or themes are identified from the data

Inclusion criteria and recruitment process

Study participants will consist of journal editors of biomedical journals, referring to individuals who are currently involved in the communication process between authors and peer reviewers

For peer review only - http://bmjopen.bmj.com/site/about/guidelines.xhtml

and/or bein a position to decide about the fate of manuscripts. They might also, but not necessarily, contribute to the determination of journal content and policy.

Journal editors will be contacted between October 2017 and February 2018. They will be sent an invitation email and information sheet by the lead author (KG), followed by a phone call to determine if they are interested in participating in the study. Written informed consent will be obtained from all participants prior to conducting the interviews.

Interview guide

A preliminary topic guide for the semi-structured interviews (see Table 5) has been developed, informed by the outcomes of a previously conducted scoping review of the literature (30). The topic guide was piloted on four editors to assess usefulness and meaningfulness of the questions, the ease of administration, language and length, and to refine the topic guide. It is likely that the topic guide will be refined further after conducting more interviews.

Table 5 – Draft topic guide for semi-structured interviews

**Key area of investigation**	**Topics**	**Questions and prompts**
Background information	- Explore personal background<br>- Level of experience<br>- Own roles and tasks as an editor	• Tell me about your journal and the job you have.<br>• How long have you been in this position?<br>**Prompt**: percentage of time devoted to editorial duties (e.g., part time, full time)<br>• What are your current responsibilities?<br>• Did you hold any other positions in the same field before your current position? If yes, what were your responsibilities then?
Journal set-up	- Explore journal set-up	• Tell me about your journal - how does it work?<br>Prompt: availability of editorial support staff<br>• How does the peer review process work in your journal?<br>• What do you do within the process?
Opinion on peer reviewers role and tasks	- Roles and tasks of peer reviewers<br>- Expectations	• What do you expect from peer reviewers in terms of their roles and tasks?<br>• How do you let your reviewers know what you expect from them?<br>**Prompt** on whatever has not been mentioned:<br>• Attitudes and beliefs (about role and tasks)<br>• Organisational expectations (about role and tasks)<br><br>• Can you tell me about a specific situation when you were not satisfied with a review or with a peer reviewer?<br>• What did you do in that a situation?<br>**Prompt**: looks for factors other than being late with a

For peer review only - http://bmjopen.bmj.com/site/about/guidelines.xhtml

		review, or not doing a review once you they have accepted it<br><br>• Can you tell me about a situation when you were exceptionally satisfied with a review or with a peer reviewer?<br>• Were there situations (in regards to the roles and task of reviewers) when you disagreed with the other editors you work with? What about? What happened?<br>• What about other journals, do roles and tasks differ among journals in your field?<br>**Prompt**: If yes (i.e. differences exist), then:<br>• How does this affect the process?<br>• How does it affect your communication?<br>• How do you negotiate those differences? Does it matter?
Communication between editors, peer reviewers and authors	- Communication between the three parties<br>- Potential conflicts<br>- Power	• Can you describe your experience of the communication process between editors, authors and peer reviews?<br>• How do you communicate with authors and peer reviewers?<br>• Can you give me some specific examples of situations where this communication is challenging?<br><br>**Prompt**:<br>What are potential conflicts?<br>When do disagreements arise?<br>What happens if there is disagreement between peer reviewers?
Conclusion	- Snowballing<br>- Documents<br>- Final comments	• Is there anybody else whom you think I should speak to?<br>• Any articles/documents I can access/should look at?<br>• Any final comments? Is there anything else that you think is important to mention?

Data collection and recording

All interviews will be conducted by the principal researcher (KG) either face-to-face or by phone or online call (e.g. Skype or conferencing system), according to the circumstances and preferences of the interviewees.

With the permission of the participants, interviews will be audio-recorded and notes will be taken.

Interviewees will be asked if they could be contacted again if further clarification is needed.

Based on the pilot interviews it is anticipated that interviews will take around 30 minutes to complete.

Data analysis

Data will be analysed using Braun and Clarke's six phase thematic analysis (TA)described as "a method for identifying, analysing and reporting patterns (themes) within data" (21). This analytic

For peer review only - http://bmjopen.bmj.com/site/about/guidelines.xhtml

framework assumes that truth can be accessed through language, but that accounts and experiences are socially mediated (31).

It is not bound to any pre-existing theoretical framework, therefore it offers relative theoretical independence and compatibility with various approaches which is compatible with pragmatist position that we subscribe to (32). TA has also been described as a more accessible form of analysis compared to other approaches that requires less detailed theoretical and technical knowledge, and is therefore particularly suitable for the lead researcher (KG) of this study who is at an early stage of her qualitative research experience (21). The principal researcher (KG) will conduct all interviews, which will be transcribed verbatim.

Data analysis will take place concurrently with data collection in an iterative cycle. This serves two purposes; firstly, it will help to further refine the topic guide, and allow the interviewer (KG) to reflect with the senior investigator (DH) on her own interviewing technique and style for subsequent interviews. Secondly, it will help the researchers to determine when saturation occurs.

The six phases of TA analysis consist of: familiarizing with the data, generating initial codes, searching for themes, reviewing themes, defining and naming themes, producing the report.

The first phase will start by familiarizing with the data - re-reading each transcript at least twice and noting down initial ideas.

In the second phase initial codes will be generated from the subset of interviews using the inductive approach. The codes will be developed line-by-line from the interview content, focusing on the identification of both semantic (i.e. reflecting the explicit content) and latent (i.e. reflect the implicit content) features (21). In order to ensure consistency a code manual will be developed. These codes will be then applied to subsequent interviews with sensitivity to the possibility of new emerging codes that will be added to the code manual and applied to the entire dataset in an iterative manner. All coding will be performed by both researchers KG and DH to increase credibility of the analysis. The qualitative data analysis software NVivo v.11 will be used to aid data management and analysis (i.e. indexing of coding and transcripts).

In the third phase the codes will be clustered into potential themes to give an indication of their prevalence for the assessment of (code or meaning) saturation, and into a preliminary thematic map displaying the main themes.

The fourth phase will consist of reviewing themes and will be divided into two stages; the reviewing and refining of the data at the level of the coded data extracts, and subsequently at the level of the entire data set. These two stages will lead to the generation of a thematic map of the analysis.

The aim of the next phase will be to definitively define the scope and content of each relevant theme and precisely name them. This will involve debriefing between the study team. Debriefing with an outside expert (on peer review in biomedical journals) as suggested by King (33) will be conducted to ensure that themes are sufficiently clear to someone outside of the immediate research team.

For peer review only - http://bmjopen.bmj.com/site/about/guidelines.xhtml

After the establishment of the final themes, the last phase will consist of writing up the study findings as a journal article. Direct quotes will be used to illustrate specific points of interpretation and the extraction of themes. All themes and subthemes will be presented in the result section and discussed in the light of existing literature.

Securing study quality

The most widely used criteria for evaluating qualitative analysis are those developed by Lincoln and Guba (34), who introduced the concept of 'trustworthiness' to parallel the conventional quantitative assessment criteria of validity and reliability. Trustworthiness is determined by applying the concepts of credibility, transferability, dependability and conformability to qualitative research. Credibility corresponds to the concept of validity, whereby researchers seek to ensure that a study

measures what it is actually intended to measure. Transferability corresponds to external validity, or the extent to which the research can be transferred to other contexts. Dependability corresponds with reliability, or whether the research process is methodologically consistent and correct, whether the research questions are clear and logically connected to the research purpose and design, and whether findings are consistent and repeatable. Confirmability is concerned with establishing that the researcher's interpretations and findings are clearly derived from the data, requiring the researcher to demonstrate how conclusions and interpretations have been reached (Tobin & Begley, 2004).

In order to establish trustworthiness in this research, the step-by-step approach proposed by Nowell et al. – which provides a detailed description of how to conduct a trustworthy TA – will be followed (35). These authors use the criteria by Lincoln and Guba and show how these can be achieved throughout the 6 phases of TA.

We will use reporting guidelines for reporting qualitative research to provide detailed reporting of methods used (36).

Patient and Public Involvement

There was no patient or public involvement in this research project.

Discussion

This research has multiple potential uses. As a standalone research piece it will generate context-based information from journal editors' perspectives that will help to provide insight into the communication patterns within biomedical journals, including differences and similarities across biomedical journals. It is also embedded within a larger project that will inform the analysis of peer reviewer reports.

The study findings can further be used to inform biomedical journal policies and develop training courses for peer reviewers and journal editors.

For peer review only - http://bmjopen.bmj.com/site/about/guidelines.xhtml

Ethics and dissemination

This project has been evaluated and approved by the University of Split, Medical School Ethics Committee (2181-198-03-04-17-0029) in May 2017.

Interviewees will receive an information sheet about the research and a consent form before the interview. The information letter includes details on the maintenance of anonymity and confidentiality throughout the research process. Prior to the interview, information from the information sheet and consent form will be reiterated verbally, and interviewees will be asked to consent to participation and recording of their interviews. Participants will be able to choose not to be directly quoted in any publications resulting from the study.

Findings will be disseminated through a publication in a peer-reviewed journal and presentations at academic conferences and other meetings.

Acknowledgements

The authors would like to thank Professor Erik Cobo for providing advice during the writing of this protocol and Dr Sara Schroter (BMJ) and Dr Elizabeth Moylan (BMC) for providing guidance and help on the recruitment strategy of interviewees.

Contributors All authors have made substantive intellectual contributions to the development of this protocol. KG conceptualized the study approach and led the writing of the manuscript. DH led the supervision of the manuscript preparation. DH was involved in developing the study questions and design and provided detailed comments on earlier drafts.

Funding This project was supported by the European Union's Horizon 2020 research and innovation programme under the Marie Sklodowska-Curie grant agreement No 676207. The funders had no role in the study design, data collection and analysis, decision to publish, or preparation of the manuscripts.

Competing interests At the time of the submission of this protocol KG conducted a secondment at the BMJ.

For peer review only - http://bmjopen.bmj.com/site/about/guidelines.xhtml

References

1. Overbeke J, Wager E. 3: The state of evidence: what we know and what we don't know about journal peer review. JAMA. 1994;272:79–174.

2. Galipeau J, Barbour V, Baskin P, Bell-Syer S, Cobey K, Cumpston M, et al. A scoping review of competencies for scientific editors of biomedical journals. BMC Med. 2016 Feb 2;14:1–17.

3. Moher D, Galipeau J, Alam S, Barbour V, Bartolomeos K, Baskin P, et al. Core competencies for scientific editors of biomedical journals: consensus statement. BMC Med. 2017 Sep 11;15(1):167.

4. Chauvin A, Ravaud P, Baron G, Barnes C, Boutron I. The most important tasks for peer reviewers evaluating a randomized controlled trial are not congruent with the tasks most often requested by journal editors. BMC Med. 2015;13(1):1.

5. Smith R. Peer review: a flawed process at the heart of science and journals. J R Soc Med [Internet]. 2006;99. Available from: http://dx.doi.org/10.1258/jrsm.99.4.178

6. Rennie D. Make peer review scientific. Nature. 2016;535(7610):31–3.

7. Silva PUKD, Vance CK. Preserving the Quality of Scientific Research: Peer Review of Research Articles. In: Scientific Scholarly Communication [Internet]. Springer International Publishing; 2017 [cited 2017 Apr 18]. p. 73–99. (Fascinating Life Sciences). Available from: http://link.springer.com/chapter/10.1007/978-3-319-50627-2_6

8. Lee CJ, Sugimoto CR, Zhang G, Cronin B. Bias in peer review. J Am Soc Inf Sci Technol. 2013 Jan 1;64(1):2–17.

9. Jefferson T, Rudin M, Brodney Folse S. Editorial peer review for improving the quality of reports of biomedical studies. Cochrane Database Syst Rev. 2007;2.

10. Bruce R, Chauvin A, Trinquart L, Ravaud P, Boutron I. Impact of interventions to improve the quality of peer review of biomedical journals: a systematic review and meta-analysis. BMC Med. 2016;14(1):85.

11. Altman DG, Schulz KF, Moher D. The revised CONSORT statement for reporting randomized trials: explanation and elaboration. Ann Intern Med [Internet]. 2001;134. Available from: http://dx.doi.org/10.7326/0003-4819-134-8-200104170-00012

12. Hojat M, Gonnella JS, Caelleigh AS. Impartial judgment by the "gatekeepers" of science: fallibility and accountability in the peer review process. Adv Health Sci Educ Theory Pract. 2003;8(1):75–96.

13. Kassirer JP, Campion EW. Peer review: crude and understudied, but indispensable. Jama. 1994;272(2):96–7.

14. King CR, McGuire DB, Longman AJ, Carroll-Johnson RM. Peer review, authorship, ethics, and conflict of interest. Image J Nurs Sch. 1997 May;29(2):163–7.

For peer review only - http://bmjopen.bmj.com/site/about/guidelines.xhtml

15. Horn SA. The Social and Psychological Costs of Peer Review: Stress and Coping With Manuscript Rejection. J Manag Inq. 2016 Jan 1;25(1):11–26.

16. Bedeian AG. Peer Review and the Social Construction of Knowledge in the Management Discipline. Acad Manag Learn Educ. 2004 Jun 1;3(2):198–216.

17. Lipworth WL, Kerridge IH, Carter SM, Little M. Journal peer review in context: A qualitative study of the social and subjective dimensions of manuscript review in biomedical publishing. Soc Sci Med. 2011 Apr;72(7):1056–63.

18. Lipworth W, Kerridge I. Shifting Power Relations and the Ethics of Journal Peer Review. Soc Epistemol. 2011 Jan 1;25(1):97–121.

19. Shashok K. Standardization vs Diversity: How Can We Push Peer Review Research Forward? Medscape Gen Med. 2005 Feb 16;7(1):11.

20. Yardley L, Bishop F. Mixing Qualitative and Quantitative Methods: A Pragmatic Approach. In: Willig C, Stainton-Rogers W, editors. Qualitative Research in Psychology. Thousand Oaks (CA): SAGE Publication Ltd.; p. 352–69.

21. Braun V, Clarke V. Using thematic analysis in psychology. Qual Res Psychol. 2006;3(2):77–101.

22. Patton M. Qualitative Research and Evaluation Methods , 209-339. 2002;

23. Projet MiRoR | An innovative and ambitious joint doctoral training programme [Internet]. [cited 2017 May 2]. Available from: http://miror-ejd.eu/

24. Rennie D, Flanagin A, Godlee F, Bloom T. The Eighth International Congress on Peer Review and Biomedical Publication: A Call for Research. JAMA. 2015 May 26;313(20):2031–2.

25. Fox CW, Burns CS, Meyer JA. Editor and reviewer gender influence the peer review process but not peer review outcomes at an ecology journal. Funct Ecol. 2016;30(1):140–53.

26. Morgan DL. Sample size. Sage Encycl Qual Res Methods. 2008;798.

27. Marshall B, Cardon P, Poddar A, Fontenot R. Does Sample Size Matter in Qualitative Research?: A Review of Qualitative Interviews in is Research. J Comput Inf Syst. 2013 Sep 1;54(1):11–22.

28. Hennink MM, Kaiser BN, Marconi VC. Code Saturation Versus Meaning Saturation: How Many Interviews Are Enough? Qual Health Res. 2016;1049732316665344.

29. Fugard AJB, Potts HWW. Supporting thinking on sample sizes for thematic analyses: a quantitative tool. Int J Soc Res Methodol. 2015 Nov 2;18(6):669–84.

For peer review only

For peer review only - http://bmjopen.bmj.com/site/about/guidelines.xhtml

30. Glonti K, Cauchi D, Cobo E, Boutron I, Moher D, Hren D. A scoping review protocol on the roles and tasks of peer reviewers in the manuscript review process in biomedical journals. BMJ Open. 2017 Oct 1;7(10):e017468.

31. Madill A, Jordan A, Shirley C. Objectivity and reliability in qualitative analysis: realist, contextualist and radical constructionist epistemologies. Br J Psychol Lond Engl 1953. 2000 Feb;91 (Pt 1):1–20.

32. Creswell JW, Poth CN. Qualitative inquiry and research design: Choosing among five approaches. Sage publications; 2017.

33. King N. Using templates in the thematic analysis of text. Essent Guide Qual Methods Organ Res. 2004;2:256–70.

34. Lincoln Y, Guba E. Naturalistic inquiry. Newburry Park. 1985;

35. Nowell LS, Norris JM, White DE, Moules NJ. Thematic Analysis: Striving to Meet the Trustworthiness Criteria. Int J Qual Methods. 2017;16(1):1609406917733847.

36. Tong A, Sainsbury P, Craig J. Consolidated criteria for reporting qualitative research (COREQ): a 32-item checklist for interviews and focus groups. Int J Qual Health Care. 2007 Dec 1;19(6):349–57.

For peer review only

For peer review only - http://bmjopen.bmj.com/site/about/guidelines.xhtml

Appendix 1. Sample size calculation

```
sampSizeForQual = function(Power, ThemePrevalence, NumInstances) { qnbinom(Power, size =
NumInstances, prob = ThemePrevalence) + NumInstances

}

sampSizeForQual(0.9, 0.1, 2)

[1] 38
```

Code run via R-Fiddle (httm://www.r-fiddle.org)

For peer review only

The editors' perspectives on the peer review process in biomedical journals: Protocol for a qualitative study

Journal:	*BMJ Open*
Manuscript ID	bmjopen-2017-020568.R2
Article Type:	Protocol
Date Submitted by the Author:	12-Jul-2018
Complete List of Authors:	Glonti, Ketevan; Sveuciliste u Splitu, School of Humanities and Social Sciences; Paris Descartes University, Sorbonne Paris Cité, INSERM, U1153 Epidemiology and Biostatistics Sorbonne Paris Cité Research Center (CRESS), Methods of therapeutic evaluation of chronic diseases Team (METHODS)<br>Hren, Darko; University of Split, School of Humanities and Social Sciences
**Primary Subject Heading**:	Medical publishing and peer review
Secondary Subject Heading:	Qualitative research
Keywords:	peer review, QUALITATIVE RESEARCH, scientific publishing

SCHOLARONE™
Manuscripts

The editors' perspectives on the peer review process in biomedical journals: Protocol for a qualitative study

Authors: Ketevan Glonti[1, 2], Darko Hren[1]

[1] University of Split, School of Humanities and Social Sciences, Split, Croatia. kglonti@unist.hr, dhren@unist.hr

[2] INSERM, U1153 Epidemiology and Biostatistics Sorbonne Paris Cité Research Center (CRESS), Methods of therapeutic evaluation of chronic diseases Team (METHODS), Paris, F-75014 France; Paris Descartes University, Sorbonne Paris Cité, France.

Corresponding author: Ketevan Glonti, Poljička 35, 21000 Split, Croatia, kglonti@unist.hr
Tel: +385 976324635

For peer review only

Abstract

Introduction: Despite dealing with scientific output and potentially having an impact on the quality of research published, the manuscript peer review process itself has at times been criticised for being 'unscientific'. Research indicates that there are social and subjective dimensions of the peer review process that contribute to this perception, including how key stakeholders - namely authors, editors and peer reviewers - communicate. In particular, it has been suggested that the expected roles and tasks of stakeholders need to be more clearly defined and communicated if the manuscript review process is to be improved. Disentangling current communication practices, and outlining the specific roles and tasks of the main actors, might be a first step towards establishing the design of interventions that counterbalance social influences on the peer review process.

The purpose of this article is to present a methodological design for a qualitative study exploring the communication practices within the manuscript review process of biomedical journals from the journal editors' point of view.

Methods and analysis: Semi-structured interviews will be carried out with editors of biomedical journals between October 2017 and February 2018. A heterogeneous sample of participants representing a wide range of biomedical journals will be sought through purposive maximum variation sampling, drawing from a professional network of contacts, publishers, conference participants, and snowballing.

Interviews will be thematically analysed following the method outlined by Braun and Clarke. The qualitative data analysis software NVivo v11 will be used to aid data management and analysis.

Ethics and dissemination: This research project was evaluated and approved by the University of Split, Medical School Ethics Committee (2181-198-03-04-17-0029) in May 2017. Findings will be disseminated through a publication in a peer-reviewed journal and presentations during conferences.

For peer review only - http://bmjopen.bmj.com/site/about/guidelines.xhtml

Keywords: Peer review, Biomedical publishing, Scientific journal publishing, Qualitative research

Strengths and limitations of this study

- Qualitative analysis of interview data from a wide range of editors of biomedical journals will allow an in-depth understanding of the communication practices prevailing within biomedical journals.
- Quality assurance will be employed throughout data collection and analysis to ensure traceability and verification.
- Journal editors of a selection of biomedical journals will be interviewed; therefore, research findings cannot directly be extrapolated to all biomedical journals and other scientific fields.

Introduction

Most journals in the biomedical field implement a pre-publication process which primarily involves the interaction of three key stakeholders around an academic research manuscript: journal editors, peer reviewers and authors. This process, typically referred to as 'peer review', is strongly embedded in the field of biomedical publishing and in its broadest sense refers to the evaluation of manuscripts submitted for publication by researchers from the same or related areas of expertise. Thus far, there is no universal definition of 'peer review', and its specific objectives are not clearly defined (1). Concurrently, the roles, tasks and core competencies expected of peer reviewers and editors have not been formally established and both groups operate largely without formal training (2,3). A study that aimed to identify all tasks that are expected of peer reviewers by journal editors when evaluating a manuscript reporting a randomized controlled trial (RCT) found that the most important tasks in peer review, as perceived by peer reviewers evaluating RCTs, were not congruent with the tasks most often requested by journal editors in their guidelines to reviewers (4). These differences illustrate the need to clarify the roles and tasks of peer reviewers.

The peer review process has at times been criticised for being 'unscientific' (5,6). Despite dealing with scientific output that potentially leads to changes in clinical practice, the process itself is not without potential biases - including prestige or association bias, gender bias, confirmation bias, conservatism, bias against interdisciplinary research, publication bias, language bias, and conflict of interest (7,8).

In light of this criticism, there have been several attempts to improve the peer review process and the quality of peer reviewer reports in the biomedical field (9). A recent systematic review evaluating the impact of interventions to improve the quality of peer review for biomedical publications (10). identified 25 strategies that have been implemented, including training interventions; use of checklists (such as Consort (11)); addition of specific experts (i.e. statistical peer reviewers); the introduction of open peer review (i.e., peer reviewers informed that their identity would be revealed) or blinded peer review (i.e., peer reviewers blinded to author names and affiliation); and interventions to increase the speed of the peer review process. The authors of the systematic review refrain from providing recommendations regarding the wider

For peer review only - http://bmjopen.bmj.com/site/about/guidelines.xhtml

implementation of the identified interventions due to concerns about their methodological quality, small sample size and applicability. Other researchers have argued that most of the approaches outlined above fail to compensate for potential biases, and point out that any success so far has been limited (12).

Researchers have argued that limited success of interventions might be due to the underlying nature of peer review, which has been described as an intellectual process that encompasses objective and subjective elements (13). Editors and peer reviewers bring a diverse mix of skills, preferences, and intellectual idiosyncrasies to the task (14). At times, these may result in subjective judgements of manuscript quality. Peer review has also been described as an "inherently human phenomenon" that is underpinned by social dimensions (15,16). A qualitative study of the social and subjective dimensions of manuscript peer review in biomedical publishing concluded that biomedical manuscript review *"is a highly social and subjective process driven by communal as well as scientific goals, and influenced by reviewers' and editors' sense of their own authority, power, and moral responsibility, as well as by unavoidable prejudice and intuition."* (17).

Our broader research framework aims to generate an understanding of the communication practices within the editorial and manuscript peer review process in biomedical research. Disentangling current communication practices for a range of biomedical journals, and outlining the specific roles and tasks of the main actors might be a first step towards establishing the design of interventions that counterbalance social influences on the peer review process. In this study, we aim to identify and characterize the roles and tasks of the different actors in the process of peer review from the perspective of journal editors.

Our specific objectives are:

1) To examine biomedical editors' experiences of their interactions with peer reviewers and authors
2) To characterize journal editors' perspectives, expectations, understandings and perceptions regarding the roles and tasks of peer reviewers

Methods and analysis

Qualitative approach and research paradigm

Given its underlying social and subjective dimensions (17,18), the need for more qualitative research into the peer review process within the biomedical field has been recognized for some time (1). However, to date, most such research has been overwhelmingly quantitative in nature (19).

Drawing on a pragmatist epistemological position that the aim of inquiry cannot be independent from human experience (20), we considered a qualitative approach to be best suited to answer our research question. The expectations, understandings, perceptions and thoughts of journal editors are largely intangible aspects that cannot be unpacked using predefined categories or viewed independently from the purposes of the peer review process itself.

For peer review only - http://bmjopen.bmj.com/site/about/guidelines.xhtml

The use of qualitative interviews will enable participants to speak freely and at length about such aspects, thus providing rich data embedded in personal experiences and practices.
Data will be analysed using thematic analysis as described by Braun and Clarke primarily because of the method's flexibility and epistemological assumptions, that are compatible with a pragmatist approach (21).
Study participants will be offered the possibility of conducting the interview either face-to-face or by phone/conferencing system, according to personal preference and availability. This will also enable the interviewing of people in geographically distant locations.

Study sample and recruitment

We will use maximum variation purposive sampling to recruit a heterogeneous study sample of biomedical journal editors, allowing us to select editors with different characteristics that we anticipate may influence their perspectives. This sampling method enables conceptual exploration using the characteristics of individuals and journals as the basis of selection in order to reflect the diversity and breadth of the sample population, rather than achieving population representativeness (22).
Participants will be recruited through different sources. The study recruitment pathway is shown in Table 1.

Table 1. Study recruitment pathways

**Source of participants**	**Sampling**
Existing professional networks	Purposive/Snowballing
Two research publishers	Purposive/Snowballing
International Congress on Peer Review and Scientific Publication	Purposive/Snowballing

Initially, interviewees will be drawn from a professional network of contacts (members of the Methods in Research on Research (MiRoR) project (23)) who are journal editors. Four editors will be interviewed for piloting purposes and requested to recommend additional journal editors whom the lead investigator can interview.

The research publishers BMC (part of Springer Nature) and BMJ are partners of the MiRoR project and this partnership will be used to recruit interviewees. Editors-in chief operating within the BMJ Publishing Group will be contacted by the lead researcher (KG) via email, provided with study details, and asked to either participate themselves or recommend suitable journal editors who can be contacted instead. One follow-up email will be sent after two weeks to non-respondents.

Due to a different standard operating procedure a different recruitment strategy will be employed at BMC. The publishers' communication manager will communicate with all editors via internal

For peer review only

mail, introduce the lead researcher and the research, and encourage them to respond if interested in participating.

Concurrently, the conference participation lists from the Eighth International Congress on Peer Review and Scientific Publication (24) will be reviewed and potential interviewees will be contacted via email by the lead researcher. One follow-up email will be sent to non-respondents after two weeks.

Following the maximum variation sampling strategy, journal editors who agree to be interviewed will be categorized using the characteristics presented in Table 2 some of which have been shown to influence the peer review process (e.g. gender) (25).
This step will help to determine the characteristics that are under-represented, and inform the sampling strategy for identification of further participants in such a way as to maximize the diversity of interviewees.

Table 2 – Sample characteristics

**Criteria**	**Characteristics**
Demographic characteristics	• Gender<br>• Editorial experience<br>• Editors' geographic location
Journal characteristics	• Journal specialty (e.g. Clinical, Public Health)<br>• Impact factor (journals with or without impact factor)<br>• Peer review practices (closed peer review, open peer review, post-publication peer review)<br>• Publisher (medical publishing companies, independent publisher/university)<br>• Open access, paywall<br>• Size (editorial team)

Lastly, the journal editor identification process will be supplemented through snowball sampling (26). At the end of each interview, interviewees will be asked to recommend other editors whose experiences might be relevant to the study and who would potentially be interested in contributing to this study. These steps are expected to lead to recommendations that optimize sample variation.

For peer review only - http://bmjopen.bmj.com/site/about/guidelines.xhtml

Saturation

Saturation is a core guiding principle to determine sample sizes in qualitative research, yet few qualitative studies report in detail on the parameters that influenced saturation in their studies (27). In this study we will adopt the seven parameters outlined by Hennink et al. that influence saturation (28) to establish our sample size determinants and demonstrate the grounds upon which saturation will be assessed and achieved, thereby justifying the final sample size. The parameters of saturation and sample size for our study are outlined in Table 3. According to Hennink et al. the sample size is determined by the combined influence of all parameters rather than any single parameter alone. In our case some parameters indicate a smaller sample for saturation and others suggest a larger sample, suggesting the need for an intermediate sample size.

The first parameter is the *purpose* of the study, which in this case is to capture themes from the data using the thematic analysis method. The second parameter is *population.* For the purposes of our study we want do grasp as wide variety of biomedical editors as possible and will thus obtain a heterogeneous sample. This parameter will be satisfied by interviewing journal editors with different characteristics (i.e. demographic characteristics, journal specialty and journal characteristics). Our *data collection strategy* will be iterative, involving continual data collection until a sample covering wide variety of experiences and viewpoints has been achieved. We aim to collect *thick data* in order to provide deep and rich insights and capture explicit and concrete codes as well as conceptual codes that capture subtle issues. Our *codebook will be emerging including* a broad range of codes, including explicit, subtle, and conceptual codes.

Lastly, the *saturation goal and focus* of our study is to achieve data saturation i.e. the point where no new issues or themes are identified from the data (28).

Although the process of reaching saturation cannot be meaningfully quantified in advance and involves an iterative approach until saturation is obtained, we used a recently developed quantitative method to offer an initial estimate of expected number of participants in our study. Following the approach suggested by Fugard and Potts (29) of estimating sample size required to achieve code saturation for studies that use thematic analysis, we calculated that we would need a sample size of at least 38 participants to detect, with 90% power, two instances of a theme with 10% prevalence. Appendix 1 shows the details of the calculation. This is in line with our previously hypothesized number of participants. Therefore, whilst our core approach to data collection strategy will be iterative, involving continual data collection until saturation is reached, we anticipate around 40 participants to be sufficient to provide us with meaningful information to answer our research questions, in line with similar studies (17).

Table 3 – Parameters of saturation and determinants of sample size for our study

**Parameters**	**Sample size determinant for each parameter**	**Determinant definition**
Purpose	Capture themes	The thematic analysis method will be used to

For peer review only - http://bmjopen.bmj.com/site/about/guidelines.xhtml

		identify themes and patterns of meanings across the dataset in relation to the research question
Population	Heterogeneous	Journal editors with different characteristics (i.e. demographic characteristics, journal discipline and characteristics)
Sampling strategy	Iterative sampling	Iterative sampling using established networks; enlarged through snowballing
Data quality	Thick data	Experiences and opinions will be captured with the aim to provide deep and rich insights
Type of codes	Conceptual codes	Explicit and subtle
Codebook	Emerging codebook	Inductive coding derived from data content including broad range of codes
Saturation goal and focus	Data saturation	Referring to saturation as the point where no new issues or themes are identified from the data

Inclusion criteria and recruitment process

Study participants will consist of journal editors of biomedical journals, referring to individuals who are currently involved in the communication process between authors and peer reviewers and/or who are in a position to decide about the fate of manuscripts. They might also, but not necessarily, contribute to the determination of journal content and policy.

Journal editors will be contacted between October 2017 and February 2018. They will be sent an invitation email and information sheet by the lead author (KG), followed by a phone call to determine if they are interested in participating in the study. Written informed consent will be obtained from all participants prior to conducting the interviews.

Interview guide

A preliminary topic guide for the semi-structured interviews (see Table 4) has been developed, informed by the outcomes of a previously conducted scoping review of the literature (30). The topic guide was piloted on four editors to assess usefulness and meaningfulness of the questions, the ease of administration, language and length, and to refine the topic guide. It is likely that the topic guide will be refined further after conducting more interviews.

Table 4 – Draft topic guide for semi-structured interviews

**Key area of investigation**	**Topics**	**Questions and prompts**

For peer review only - http://bmjopen.bmj.com/site/about/guidelines.xhtml

Background information	- Explore personal background<br>- Level of experience<br>- Own roles and tasks as an editor	• Tell me about your journal and the job you have.<br>• How long have you been in this position?<br>**Prompt**: percentage of time devoted to editorial duties (e.g., part time, full time)<br>• What are your current responsibilities?<br>• Did you hold any other editorial position before your current position? If yes, what were your responsibilities then?
Journal set-up	- Explore journal set-up	• Tell me about your journal - how does it work? Prompt: availability of editorial support staff<br>• How does the peer review process work in your journal?<br>• What do you do within the process?
Opinion on peer reviewers role and tasks	- Roles and tasks of peer reviewers<br>- Expectations	• What do you expect from peer reviewers in terms of their roles and tasks?<br>• How do you let your reviewers know what you expect from them?<br>**Prompt** on whatever has not been mentioned:<br>• Attitudes and beliefs (about role and tasks)<br>• Organisational expectations (about role and tasks)<br><br>• Can you tell me about a specific situation when you were not satisfied with a review or with a peer reviewer?<br>• What did you do in that a situation?<br>**Prompt**: looks for factors other than being late with a review, or not doing a review once you they have accepted it<br><br>• Can you tell me about a situation when you were exceptionally satisfied with a review or with a peer reviewer?<br>• Were there situations (in regards to the roles and task of reviewers) when you disagreed with the other editors you work with? What about? What happened?<br>• What about other journals, do roles and tasks differ among journals in your field?<br>**Prompt**: If yes (i.e. differences exist), then:<br>• How does this affect the process?<br>• How does it affect your communication?<br>• How do you negotiate those differences? Does it matter?
Communication between editors, peer reviewers and authors	- Communication between the three parties<br>- Potential conflicts<br>- Power	• Can you describe your experience of the communication process between editors, authors and peer reviews?<br>• How do you communicate with authors and peer reviewers?<br>• Can you give me some specific examples of situations where this communication is challenging?

For peer review only - http://bmjopen.bmj.com/site/about/guidelines.xhtml

		**Prompt**:<br>What are potential conflicts?<br>When do disagreements arise?<br>What happens if there is disagreement between peer reviewers?
Conclusion	- Snowballing<br>- Documents<br>- Final comments	• Is there anybody else whom you think I should speak to?<br>• Any articles/documents I can access/should look at?<br>• Any final comments? Is there anything else that you think is important to mention?

Data collection and recording

All interviews will be conducted by the principal researcher (KG) either face-to-face or by phone or online call (e.g. Skype or conferencing system), according to the circumstances and preferences of the interviewees.

With the permission of the participants, interviews will be audio-recorded and notes will be taken.

Interviewees will be asked if they could be contacted again if further clarification is needed.

Based on the pilot interviews it is anticipated that interviews will take around 30 minutes to complete.

Data analysis

Data will be analysed using Braun and Clarke's six phase thematic analysis (TA) described as "a method for identifying, analysing and reporting patterns (themes) within data" (21). This analytic framework assumes that truth can be accessed through language, but that accounts and experiences are socially mediated (31).

It is not bound to any pre-existing theoretical framework, therefore it offers relative theoretical independence and compatibility with various approaches which is compatible with pragmatist position that we subscribe to (32). TA has also been described as a more accessible form of analysis compared to other approaches that requires less detailed theoretical and technical knowledge, and is therefore particularly suitable for the lead researcher (KG) of this study who is at an early stage of her qualitative research experience (21). The principal researcher (KG) will conduct all interviews, which will be transcribed verbatim.

Data analysis will take place concurrently with data collection in an iterative cycle. This serves two purposes; firstly, it will help to further refine the topic guide, and allow the interviewer (KG) to reflect with the senior investigator (DH) on her own interviewing technique and style for subsequent interviews. Secondly, it will help the researchers to determine when saturation occurs.

The six phases of TA analysis consist of: familiarizing with the data, generating initial codes, searching for themes, reviewing themes, defining and naming themes, producing the report.

The first phase will start by familiarizing with the data - re-reading each transcript at least twice and noting down initial ideas.

In the second phase initial codes will be generated from the subset of interviews using the inductive approach. The codes will be developed line-by-line from the interview content, focusing

For peer review only - http://bmjopen.bmj.com/site/about/guidelines.xhtml

on the identification of both semantic (i.e. reflecting the explicit content) and latent (i.e. reflect the implicit content) features (21). In order to ensure consistency a code manual will be developed by both researchers (KG and DH) to increase credibility. These codes will be then applied to subsequent interviews with sensitivity to the possibility of new emerging codes that will be added to the code manual and applied to the entire dataset in an iterative manner. Coding will be performed by both researchers KG and DH to increase credibility of the analysis. The qualitative data analysis software NVivo v.11 will be used to aid data management and analysis (i.e. indexing of coding and transcripts).

In the third phase the codes will be clustered into potential themes to give an indication of their prevalence for the assessment of (code or meaning) saturation, and into a preliminary thematic map displaying the main themes.

The fourth phase will consist of reviewing themes and will be divided into two stages; the reviewing and refining of the data at the level of the coded data extracts, and subsequently at the level of the entire data set. These two stages will lead to the generation of a thematic map of the analysis.

The aim of the next phase will be to definitively define the scope and content of each relevant theme and precisely name them. This will involve debriefing between the study team. Debriefing with an outside expert (on peer review in biomedical journals) as suggested by King (33) will be conducted to ensure that themes are sufficiently clear to someone outside of the immediate research team.

After the establishment of the final themes, the last phase will consist of writing up the study findings as a journal article. Direct quotes will be used to illustrate specific points of interpretation and the extraction of themes. All themes and subthemes will be presented in the result section and discussed in the light of existing literature.

Securing study quality

The most widely used criteria for evaluating qualitative analysis are those developed by Lincoln and Guba (34), who introduced the concept of 'trustworthiness' to parallel the conventional quantitative assessment criteria of validity and reliability. Trustworthiness is determined by applying the concepts of credibility, transferability, dependability and conformability to qualitative research. Credibility corresponds to the concept of validity, whereby researchers seek to ensure that a study measures what it is actually intended to measure. Transferability corresponds to external validity, or the extent to which the research can be transferred to other contexts. Dependability corresponds with reliability, or whether the research process is methodologically consistent and correct, whether the research questions are clear and logically connected to the research purpose and design, and whether findings are consistent and repeatable. Confirmability is concerned with establishing that the researcher's interpretations and findings are clearly derived from the data, requiring the researcher to demonstrate how conclusions and interpretations have been reached (35).

For peer review only - http://bmjopen.bmj.com/site/about/guidelines.xhtml

For peer review only

In order to establish trustworthiness in this research, the step-by-step approach proposed by Nowell et al. – which provides a detailed description of how to conduct a trustworthy TA – will be followed (36). These authors use the criteria by Lincoln and Guba and show how these can be achieved throughout the 6 phases of TA.
We will use reporting guidelines for reporting qualitative research to provide detailed reporting of methods used (37).

Patient and Public Involvement

There was no patient or public involvement in this research project.

Discussion

This research has multiple potential uses. As a standalone research piece it will generate context-based information from journal editors' perspectives that will help to provide insight into the communication patterns within biomedical journals, including differences and similarities across biomedical journals. It is also embedded within a larger project that will inform the analysis of peer reviewer reports.
The study findings can further be used to inform biomedical journal policies and develop training courses for peer reviewers and journal editors.

Ethics and dissemination

This project has been evaluated and approved by the University of Split, Medical School Ethics Committee (2181-198-03-04-17-0029) in May 2017.
Interviewees will receive an information sheet about the research and a consent form before the interview. The information letter includes details on the maintenance of anonymity and confidentiality throughout the research process. Prior to the interview, information from the information sheet and consent form will be reiterated verbally, and interviewees will be asked to consent to participation and recording of their interviews. Participants will be able to choose not to be directly quoted in any publications resulting from the study.

Findings will be disseminated through a publication in a peer-reviewed journal and presentations at academic conferences and other meetings.

Acknowledgements

The authors would like to thank Professor Erik Cobo for providing advice during the writing of this protocol and Dr Sara Schroter (BMJ) and Dr Elizabeth Moylan (BMC) for providing guidance and help on the recruitment strategy of interviewees.

Contributors All authors have made substantive intellectual contributions to the development of this protocol. KG conceptualized the study approach and led the writing of the manuscript. DH led

For peer review only - http://bmjopen.bmj.com/site/about/guidelines.xhtml

For peer review only

the supervision of the manuscript preparation. DH was involved in developing the study questions and design and provided detailed comments on earlier drafts.

Funding This project was supported by the European Union's Horizon 2020 research and innovation programme under the Marie Sklodowska-Curie grant agreement No 676207. The funders had no role in the study design, data collection and analysis, decision to publish, or preparation of the manuscripts.

Competing interests At the time of the submission of this protocol KG conducted a secondment at the BMJ.

For peer review only

References

1. Overbeke J, Wager E. 3: The state of evidence: what we know and what we don't know about journal peer review. JAMA. 1994;272:79–174.

2. Galipeau J, Barbour V, Baskin P, Bell-Syer S, Cobey K, Cumpston M, et al. A scoping review of competencies for scientific editors of biomedical journals. BMC Med. 2016 Feb 2;14:1–17.

3. Moher D, Galipeau J, Alam S, Barbour V, Bartolomeos K, Baskin P, et al. Core competencies for scientific editors of biomedical journals: consensus statement. BMC Med. 2017 Sep 11;15(1):167.

4. Chauvin A, Ravaud P, Baron G, Barnes C, Boutron I. The most important tasks for peer reviewers evaluating a randomized controlled trial are not congruent with the tasks most often requested by journal editors. BMC Med. 2015;13(1):1.

5. Smith R. Peer review: a flawed process at the heart of science and journals. J R Soc Med [Internet]. 2006;99. Available from: http://dx.doi.org/10.1258/jrsm.99.4.178

6. Rennie D. Make peer review scientific. Nature. 2016;535(7610):31–3.

7. Silva PUKD, Vance CK. Preserving the Quality of Scientific Research: Peer Review of Research Articles. In: Scientific Scholarly Communication [Internet]. Springer International Publishing; 2017 [cited 2017 Apr 18]. p. 73–99. (Fascinating Life Sciences). Available from: http://link.springer.com/chapter/10.1007/978-3-319-50627-2_6

8. Lee CJ, Sugimoto CR, Zhang G, Cronin B. Bias in peer review. J Am Soc Inf Sci Technol. 2013 Jan 1;64(1):2–17.

9. Jefferson T, Rudin M, Brodney Folse S. Editorial peer review for improving the quality of reports of biomedical studies. Cochrane Database Syst Rev. 2007;2.

10. Bruce R, Chauvin A, Trinquart L, Ravaud P, Boutron I. Impact of interventions to improve the quality of peer review of biomedical journals: a systematic review and meta-analysis. BMC Med. 2016;14(1):85.

11. Altman DG, Schulz KF, Moher D. The revised CONSORT statement for reporting randomized trials: explanation and elaboration. Ann Intern Med [Internet]. 2001;134. Available from: http://dx.doi.org/10.7326/0003-4819-134-8-200104170-00012

12. Hojat M, Gonnella JS, Caelleigh AS. Impartial judgment by the "gatekeepers" of science: fallibility and accountability in the peer review process. Adv Health Sci Educ Theory Pract. 2003;8(1):75–96.

13. Kassirer JP, Campion EW. Peer review: crude and understudied, but indispensable. Jama. 1994;272(2):96–7.

14. King CR, McGuire DB, Longman AJ, Carroll-Johnson RM. Peer review, authorship, ethics, and conflict of interest. Image J Nurs Sch. 1997 May;29(2):163–7.

For peer review only - http://bmjopen.bmj.com/site/about/guidelines.xhtml

15. Horn SA. The Social and Psychological Costs of Peer Review: Stress and Coping With Manuscript Rejection. J Manag Inq. 2016 Jan 1;25(1):11–26.

16. Bedeian AG. Peer Review and the Social Construction of Knowledge in the Management Discipline. Acad Manag Learn Educ. 2004 Jun 1;3(2):198–216.

17. Lipworth WL, Kerridge IH, Carter SM, Little M. Journal peer review in context: A qualitative study of the social and subjective dimensions of manuscript review in biomedical publishing. Soc Sci Med. 2011 Apr;72(7):1056–63.

18. Lipworth W, Kerridge I. Shifting Power Relations and the Ethics of Journal Peer Review. Soc Epistemol. 2011 Jan 1;25(1):97–121.

19. Shashok K. Standardization vs Diversity: How Can We Push Peer Review Research Forward? Medscape Gen Med. 2005 Feb 16;7(1):11.

20. Yardley L, Bishop F. Mixing Qualitative and Quantitative Methods: A Pragmatic Approach. In: Willig C, Stainton-Rogers W, editors. Qualitative Research in Psychology. Thousand Oaks (CA): SAGE Publication Ltd.; p. 352–69.

21. Braun V, Clarke V. Using thematic analysis in psychology. Qual Res Psychol. 2006;3(2):77–101.

22. Patton M. Qualitative Research and Evaluation Methods , 209-339. 2002;

23. Projet MiRoR | An innovative and ambitious joint doctoral training programme [Internet]. [cited 2017 May 2]. Available from: http://miror-ejd.eu/

24. Rennie D, Flanagin A, Godlee F, Bloom T. The Eighth International Congress on Peer Review and Biomedical Publication: A Call for Research. JAMA. 2015 May 26;313(20):2031–2.

25. Fox CW, Burns CS, Meyer JA. Editor and reviewer gender influence the peer review process but not peer review outcomes at an ecology journal. Funct Ecol. 2016;30(1):140–53.

26. Morgan DL. Sample size. Sage Encycl Qual Res Methods. 2008;798.

27. Marshall B, Cardon P, Poddar A, Fontenot R. Does Sample Size Matter in Qualitative Research?: A Review of Qualitative Interviews in is Research. J Comput Inf Syst. 2013 Sep 1;54(1):11–22.

28. Hennink MM, Kaiser BN, Marconi VC. Code Saturation Versus Meaning Saturation: How Many Interviews Are Enough? Qual Health Res. 2016;1049732316665344.

29. Fugard AJB, Potts HWW. Supporting thinking on sample sizes for thematic analyses: a quantitative tool. Int J Soc Res Methodol. 2015 Nov 2;18(6):669–84.

For peer review only

For peer review only - http://bmjopen.bmj.com/site/about/guidelines.xhtml

30. Glonti K, Cauchi D, Cobo E, Boutron I, Moher D, Hren D. A scoping review protocol on the roles and tasks of peer reviewers in the manuscript review process in biomedical journals. BMJ Open. 2017 Oct 1;7(10):e017468.

31. Madill A, Jordan A, Shirley C. Objectivity and reliability in qualitative analysis: realist, contextualist and radical constructionist epistemologies. Br J Psychol Lond Engl 1953. 2000 Feb;91 (Pt 1):1–20.

32. Creswell JW, Poth CN. Qualitative inquiry and research design: Choosing among five approaches. Sage publications; 2017.

33. King N. Using templates in the thematic analysis of text. Essent Guide Qual Methods Organ Res. 2004;2:256–70.

34. Lincoln Y, Guba E. Naturalistic inquiry. Newburry Park. 1985;

35. Tobin GA, Begley CM. Methodological rigour within a qualitative framework. J Adv Nurs. 48(4):388–96.

36. Nowell LS, Norris JM, White DE, Moules NJ. Thematic Analysis: Striving to Meet the Trustworthiness Criteria. Int J Qual Methods. 2017;16(1):1609406917733847.

37. Tong A, Sainsbury P, Craig J. Consolidated criteria for reporting qualitative research (COREQ): a 32-item checklist for interviews and focus groups. Int J Qual Health Care. 2007 Dec 1;19(6):349–57.

For peer review only

For peer review only - http://bmjopen.bmj.com/site/about/guidelines.xhtml

Appendix 1. Sample size calculation

```
sampSizeForQual = function(Power, ThemePrevalence, NumInstances) { qnbinom(Power, size = NumInstances, prob = ThemePrevalence) + NumInstances

}

sampSizeForQual(0.9, 0.1, 2)

[1] 38
```

Code run via R-Fiddle (httm://www.r-fiddle.org)

For peer review only
